# Schizophrenia: An Impairment in the Capacity to Perceive Affordances

**DOI:** 10.3389/fpsyg.2017.01052

**Published:** 2017-06-28

**Authors:** Nam-Gyoon Kim, Hakboon Kim

**Affiliations:** Department of Psychology, Keimyung UniversityDaegu, South Korea

**Keywords:** schizophrenia, self-disorder, affordance, reciprocity, disembodiment

## Abstract

Phenomenological psychopathologists conceptualize schizophrenia as a self-disorder involving profound distortions of selfhood. For James Gibson, “to perceive the world is to coperceive oneself.” If the sense of self is disturbed in individuals with schizophrenia, this could also lead to disturbances in these individuals’ ability to perceive affordances, environmental properties taken with reference to the perceiver’s action capabilities (e.g., a rigid surface affording ‘walk-on-able,’ chairs ‘sit-on-able,’ and so on). To test this hypothesis, three experiments investigated schizophrenia patients’ affordance perception. Participants were presented with a photo of a common object on the computer and then asked to judge its secondary affordance (a non-designed function) in a two-choice reaction time task in Experiment 1 and in a yes/no task in Experiment 2. Schizophrenia participants performed less accurately and more slowly than controls. To rule out visual impairment as a contributing factor, in Experiment 3, participants identified physical properties (color, shape, material composition) of the objects. Schizophrenia participants were as accurate as controls and responded faster than in the previous experiments. Results suggest that the capacity to perceive affordances is likely impaired in people with schizophrenia, although the capacity to detect the object’s physical properties is kept intact. Inability to perceive affordances, those functionally significant properties of the surrounding environment, may help explain why schizophrenia patients may appear as somewhat detached from the world.

## Introduction

Schizophrenia, in which patients can exhibit psychotic episodes, bizarre behavior, and detachment from reality, is the most baffling mental disease in the field of psychiatry. Despite more than a century of intensive research, controversy persists as to how this complex disease and its perplexing symptoms should be conceptualized. Recently, proponents of phenomenological psychiatry have conceptualized schizophrenia as a disorder of self in which disembodiment is the fundamental feature of schizophrenia patients’ experience (Parnas, 2000, 2003, 2011, 2012; Sass, 2003a,b, 2014; Sass and Parnas, 2003, 2007; Cermolacce et al., 2007; Parnas and Sass, 2010; Raballo et al., 2011; Nelson et al., 2014, to name a few).

Affordance, the central concept of Gibson’s (1986/1979) ecological approach to perception and action, refers to environmental properties taken with reference to an animal. By perceiving affordances, animals become aware of functionally significant properties of the surrounding environment. For Gibson (1986/1979), affordance is a concept that “cuts across the dichotomy of subjective–objective” (p. 129). Furthermore, information for Gibson is comprised of two components, one specific to the self (proprioception) and the other specific to the environment (exteroperception) that are inseparable, like two sides of a coin (Turvey, 1992; Reed, 1996). As Gibson put it: “One perceives the environment and coperceives oneself” (p. 126). If distortions of selfhood are indeed a core feature of schizophrenia, as phenomenological psychopathologists contend, it can be conjectured that the capacity of schizophrenia patients to perceive affordances may be equally disturbed.

The present study investigated schizophrenia patients’ affordance perception and the possible consequences of compromised affordance perception to provide possible empirical validation for previous phenomenological observations and further enhance our understanding of this disorder. In the following sections, we describe Gibson’s affordance theory and our rationale for its use in this study. We then summarize phenomenological psychopathologists’ conceptualization of schizophrenia as a disorder of self.

## Theoretical Framework: Gibson’s Reciprocity-Based Theory of Perception–Action

### Animal–Environment Reciprocity

James J. Gibson’s ecological approach to perception and action is founded on the principle of reciprocity (Lombardo, 1987; Heft, 2001; Adolph and Kretch, 2015). At the ecological scale of an individual animal, the animal’s behavior is tailored with respect to its surrounding environment, whereas the environment provides behavioral opportunities to the animal. Mutually complementing each other, animals and their surrounding environments are integrated as a reciprocally interactive ecosystem.

To account for the reciprocal interaction between the animal and the environment, Gibson (1977, 1986/1979) introduced what he termed “affordances,” environmental properties taken with reference to animals. In this view, an animal encountering the surrounding environment perceives a layout of surfaces scaled relative to the animal’s anatomy and its action capabilities, rather than objective qualities (e.g., shape, size, texture, color, composition, mass, and motion) indifferent to the animal’s scale and action capabilities. For example, for humans, a flat, rigid, extended, and knee-high surface affords sitting. However, a specific chair may be sit-on-able for an adult, but only climb-on-able for a toddler. An object can have many different affordances, depending on the observer’s action capabilities and/or behavioral goals, for affordances are related, not only to the environment, but also to the observer. For example, a specific chair may also afford being used as a step stool (climb-on-able) or to bar a door. As Gibson (1986/1979) put it, “The *affordances* of the environment are what it *offers* the animal, what it *provides* or *furnishes*, either for good or ill… It implies the complementarity of the animal and the environment” (Gibson, 1986/1979, p. 127, italics original).

### Perception–Action Reciprocity

To perceive an affordance, an animal must detect the information specifying it. This information is available in the structured energy pattern ambient at any given point of observation, and is uniquely determined by the environmental layout. Gibson argued that, to detect such information, the animal must seek it actively. In the words of Gibson (1986), “We must perceive in order to move, but we must also move in order to perceive” (p. 223). In the case of vision, this involves not only using the eyes, but “the eyes in the head on the shoulders of a body that gets about” (Gibson, 1986/1979, p. 222). Whereas the available affordance information must be actively detected by the animal, reciprocally, the animal’s action is guided by perceptual information. As the animal moves, its motion uniquely transforms the ambient energy distribution in accordance with the changes in the environmental layout and the displacements of the moving observation point. This transforming energy pattern at a moving point of observation, or optic flow, in turn, is specific to the environmental layout and the animal’s movements that engendered it (Gibson, 1966, 1986/1979; Warren, 1998, 2006). For example, forward movement structures the optic flow such that all the optical elements radiate from a single point, the focus of expansion, corresponding to the actor’s movement direction. By regulating the direction of movement coincident with the focus of expansion, the animal can reach its intended target.

### Perception–Proprioception Reciprocity

As noted above, the ambient optic array at a given point of observation is structured exclusively by objects and the layout of the environment. Therefore, the structure of the optic array is specific to the facts of the animal’s surroundings. When an observer occupies a point of observation, the portion of the ambient array sampled by the observer is confined to the field of view registered by her eyes (see Figure 7.1 in Gibson, 1986/1979). This field of view, which can be portrayed as an oval window, contains various optical structures, some of which correspond to the observer’s body parts, such as the nose, lips, cheek, and limbs. As the observer moves, for example, rotating her head, those structures corresponding to her body parts transform. Because the optical transformation is produced by observer movement, the patterns of transformation are specific to the movements that produced them. For Gibson, perception of the environment and perception of the self were inseparable, always occurring together (Reed, 1996). As Gibson remarked, “One perceives the environment and coperceives oneself” (p. 126).

In the optic array, information specific to the environment is called extero-specific and information specific to the observer is termed proprio-specific. Since Sherrington (1906), it has commonly been thought that self-perception is conveyed by information from mechanoreceptors in the muscles, tendons, and joints. For Gibson, however, awareness of self can also be gained by visual information. Gibson referred to this visually based awareness of self as *visual kinesthesis*.

“Vision is *kinesthetic* in that it registers movements of the body just as much as does the muscle-joint-skin system and the inner ear system. … Visual kinesthesis goes along with muscular kinesthesis. The doctrine that vision is exteroceptive, that it obtains ‘external’ information only, is simply false. Vision obtains information about *both* the environment and the self” (Gibson, 1986/1979, p. 183, italics original).

### Affordances

For Gibson, the concept of affordance binds the reciprocal pairs (duals) of animal and environment, perception and action, (subjective) self and (objective) world, into a dynamic whole.^1^ As Gibson (1986/1979) put it,

“An affordance is neither an objective property nor a subjective property; or it is both if you like.^2^ An affordance cuts across the dichotomy of subjective–objective and helps us to understand its inadequacy. It is equally a fact of the environment and a fact of behavior. It is both physical and psychical, yet neither. An affordance points both ways, to the environment and to the observer” (p. 129).

An extensive body of research has demonstrated that human observers are capable of perceiving a variety of affordances (see Fajen et al., 2008, for review). Whether environmental layouts are perceived in body-scaled terms (Warren, 1984; Mark, 1987; Warren and Whang, 1987; Carello et al., 1989; Wraga, 1999) or action capabilities (Oudejans et al., 1996; Cesari et al., 2003), as Gibson contended, research findings to date confirm that affordances, rather than physical properties, are what animals perceive.

## Schizophrenia as a Disturbance of Minimal Self

Characterized by its diverse mental symptoms, schizophrenia is probably the most debilitating and the most conceptually challenging of all mental disorders (Arango and Carpenter, 2011). Since the introduction of operationalized diagnostic criteria (i.e., the American Psychiatric Association’s Diagnostic and Statistical Manual of Mental Disorder, DSM-5, and the World Health Organization’s International Statistical Classification of Diseases and Related Health Problems, ICD-10) and structured interviews, the reliability of the disease’s diagnosis has improved substantially (Conklin and Iacono, 2003). Advances in neuroscience and molecular genetics have further enhanced our understanding of this disability (Weinberger and Harrison, 2011, for review). Nevertheless, its etiology remains elusive (Wong and Van Tol, 2003; Insel, 2010; Jablensky, 2010).

### Schizophrenia from a Phenomenological Perspective

Because of schizophrenia’s complexity and still unidentified etiology and pathogenesis, researchers disagree as to how this disorder should be conceptualized. The present diagnostic system is grounded in the reductionist paradigm of neuroscience and biological psychiatry. This approach aims to identify the underlying cognitive and neurobiological processes for each symptom or symptom group comprising the diverse psychopathology of schizophrenia (Persons, 1986; Cahill and Frith, 1996). Proponents of phenomenological psychiatry and philosophy argue that the reductionist approach fails to recognize the illness’s first-person dimensions (Lysaker and Lysaker, 2010). Sass and Parnas (2003), in particular, contend that the disparate psychopathological symptoms of schizophrenia may be manifestations of a single phenomenological core, i.e., a disturbance of “ipseity” (*ipse* in Latin meaning “self” or “itself”), also referred to as minimal self, basic self, core self, or proto-self, which accounts for the pre-reflective (i.e., non-conceptual) and primitive level of subjective awareness, i.e., sense of ownership (i.e., an awareness of being the source of phenomenal experiences) and sense of agency (i.e., an awareness of being the agent executing one’s own actions) (Gallagher, 2000; Sass and Parnas, 2003; Zahavi, 2005; Stanghellini, 2009; Fuchs, 2010; Parnas and Sass, 2010; Nelson et al., 2014). The sources of core self distortion are thought to be two mutually interdependent processes—hyperreflexivity and diminished self-affection. Hyperreflexivity refers to a form of exaggerated self-consciousness in which tacit aspects of oneself become objectified as if they were external objects; Diminished self-affection refers to a weakening sense of being a subject of awareness (Sass and Parnas, 2003).

An individual’s awareness of his own thoughts, actions, perceptions, feelings, or pains operates at a pre-reflective (i.e., direct, immediate, implicit, non-conceptual, non-inferential, or non-reflective) level. This awareness of selfhood, as Sass and Parnas contend (Parnas, 2000, 2003, 2011, 2012; Sass, 2003a,b, 2014; Sass and Parnas, 2003; Cermolacce et al., 2007; Parnas and Sass, 2010; Raballo et al., 2011; Nelson et al., 2014), can be so profoundly disturbed in patients with schizophrenia that the distinction between self and others is altered or even vanishes. The patient’s body, normally taken for granted, begins to feel strange and unfamiliar, inviting explicit attention. As self-observation increases, aspects of the self begin to separate or detach such that “one’s arms or legs, one’s face, the feelings in the mouth or throat, the orbital housing of the eyes—even one’s speaking, thinking, or feeling” become “objectified, alien, and apart, perhaps even like the possessions of some foreign being” (Sass and Parnas, 2007, p. 432). The alienated body and aspects of one’s feeling increasingly command the individual’s introspection and intense reflection. Because the “detached” body no longer mediates between the self and the world, the patient’s normal sense of immersion in the world fades away and the world loses its practical meaning. Ultimately, the schizophrenia patient takes on the characteristics of a soulless body or a disembodied spirit^3^ (Stanghellini, 2009).

## The Present Study

As described earlier, the principle of reciprocity constitutes the conceptual foundation of Gibson’s ecological psychology. Animals are reciprocally coupled with their surrounding environments, perception with action, and (extero-)perception with proprioception. The concept of affordance theoretically merges these pairs into a dynamic whole. By perceiving the environmental affordances that offer opportunities for action, animals are kept in touch with their surroundings, that is, they are embodied.

If, as phenomenological psychopathologists (Parnas, 2000, 2003, 2011, 2012; Stanghellini and Ballerini, 2002; Sass, 2003a,b, 2014; Sass and Parnas, 2003, 2007; Uhlhaas and Mishara, 2007; Fuchs, 2009, 2010; Fuchs and Schlimme, 2009; Stanghellini, 2009; Parnas and Sass, 2010) contend, selfhood is profoundly distorted in schizophrenia, it is conceivable that reciprocities that support the dynamic interplay between an animal and its surroundings would be significantly affected. Distortions would occur, not only in the patient’s perceptions of the world, but also in his perception of self. As his awareness of his own action capabilities deteriorates, his capacity to perceive affordances would also be severely compromised, depleting his sense of embodiment in the world. It is this possibility that was explored in the present study.

Although rare, schizophrenia patients’ self-reports have contributed to phenomenological psychopathologists’ conceptualization of this disease. Articulating one’s subjective experience using normal everyday language is not easy for anyone. For schizophrenia patients, the task is even more challenging because their altered mental states make it difficult for them to maintain their thought or discourse focused on any topic (Sass, 2001). With contemporary advances in neuroscience, current understanding of schizophrenia has broadened substantially (Weinberger and Harrison, 2011, for review). Still, many issues related to this disorder and its etiology have yet to be resolved. Thus, additional insights into the subjective experience of the patient may provide a richer and more valid understanding of the psychopathology of this complex disorder. The present study aimed to demonstrate empirically phenomenological anomalies in schizophrenia patients’ perception to gain a better understanding of the disorder.

Devising an empirical study to investigate others’ subjective experience poses a significant challenge. One must also factor in the difficulties in obtaining cooperation from schizophrenia patients (Kertzman et al., 2006), whose attentional capacities are likely impaired (Fioravanti et al., 2005; Goff et al., 2011). Following the methods used by Kertzman et al. (2006) computer-based, two-alternative, forced choice tasks were employed for this research to minimize the demand on working memory, verbal or numerical ability.

The modern human environment includes numerous objects designed to be used for a certain purpose. When encountering well-designed artifacts, the designer’s intended affordances are easily recognized. However, human artifacts often provide more than one affordance because of their multiple properties (e.g., shape, size, and material composition) (Ye et al., 2009). For example, chopsticks, which were originally designed to be eating utensils, can also function as skewers, stirrers, or even drum sticks, depending on the user’s needs.

It is possible to group together diverse objects, each with a different primary affordance, under the same secondary affordance. For example, a bowl, a jam jar, a hat, or even a shoe can all support (afford) scooping water from the brook. For that reason, a secondary affordance can serve as an effective tool to assess affordance perception capacity. We combined Ye et al.’s (2009) multiple affordance research paradigm with the procedure employed by Sevos et al. (2013) to minimize the demand on working memory, verbal skills, or numerical ability. Sevos et al. (2013) asked participants (one group of schizophrenia patients and one group of controls) to press one of two response keys as fast and accurately as possible, with their left or right index finger depending on the vertical orientation of the displayed objects (upright or inverted), a classic stimulus-response compatibility task^4^ employed by Tucker and Ellis (1998). In the present study, participants were asked to judge the affordance or physical property of the displayed object depending on the experiment.

Three experiments investigated schizophrenia patients’ (non-primary) affordance perception. In the first experiment (Multiple Affordance Discrimination), participants viewed an object drawn from two different object sets, with each set having a different type of non-primary affordance, and were asked to identify its affordance, that is, to judge whether the object could be used to perform a certain function (or action) (e.g., mopping up water spilled on a table). Accuracy and reaction times of schizophrenia participants were compared to those of age-matched healthy adults. The second and third experiments served as controls. In the second experiment (Single Affordance Detection), participants were asked whether the object had a specific affordance. The first experiment had employed a two-choice reaction time task, but the second experiment employed a yes/no decision task, further simplifying task complexity. In the third experiment (Physical Property Detection), using the same procedure as in Experiment 2, participants were asked whether an object had a certain physical property (e.g., shape, color, or material composition). Based on Ye et al.’s (2009) results, in which young adults perceived non-primary affordances conveyed by various objects quite easily, we expected this task to be simple and straight-forward. However, we anticipated poorer performance by patients with schizophrenia, perhaps related to the disturbance in self-awareness that interferes with their recognition of the practical utility of objects, as reported by phenomenological psychopathologists.

## Experiment 1: Multiple Affordance Discrimination

### Participants

Twenty-six community-dwelling schizophrenia outpatients (19 males and 7 females) and 23 healthy control participants (18 males and 5 females) participated in the study. Schizophrenia participants had been diagnosed in accordance with the DSM-IV diagnostic criteria for schizophrenia (American Psychiatric Association, 1994) and were following individualized medication regimes. Schizophrenia participants were recruited from local (vocational) rehabilitation centers and mental health clinics. The control participants, who had no known family history of mental health disorder, were recruited from the university community.

For the schizophrenia group, the mean age was 41.0 ± 9.1 years, the mean years of education were 12.9 ± 2.25 years, the mean duration of schizophrenia was 18.2 ± 8.5 years, and the mean age of onset was 23.2 ± 4.4 years. For the control group, the mean age was 42.0 ± 7.45 years and the mean years of education were 13.6 ± 1.88 years. The two groups matched for age, *t*(47) = 0.42, *p* > 0.05, and education, *t*(47) = 1.24, *p* > 0.05.

One of the core features of schizophrenia is impaired cognition (Fioravanti et al., 2005, 2012; Goff et al., 2011). To exclude the possible effect of impaired cognition on task performance, we assessed each participant’s cognitive impairment using the Korean adaptation (Kwon and Park, 1989) of the Mini Mental State Examination (MMSE) (Folstein et al., 1975). For the schizophrenia group, the mean MMSE score was 28.1 ± 1.38, with five minimum scores of 26. For the control group, it was 28.5 ± 0.90, a negligible difference, *t*(47) = 1.315, *p* > 0.05.

All participants had normal or corrected-to-normal vision. Participants received a nominal ($5) fee for their participation in the experiment.

### Ethics Statement

The study was approved by the Keimyung University’s Institutional Review Board. After providing a complete description of the study to the participants, written informed consent was obtained in accordance with the Declaration of Helsinki.

### Apparatus and Materials

**FIGURE 1:**
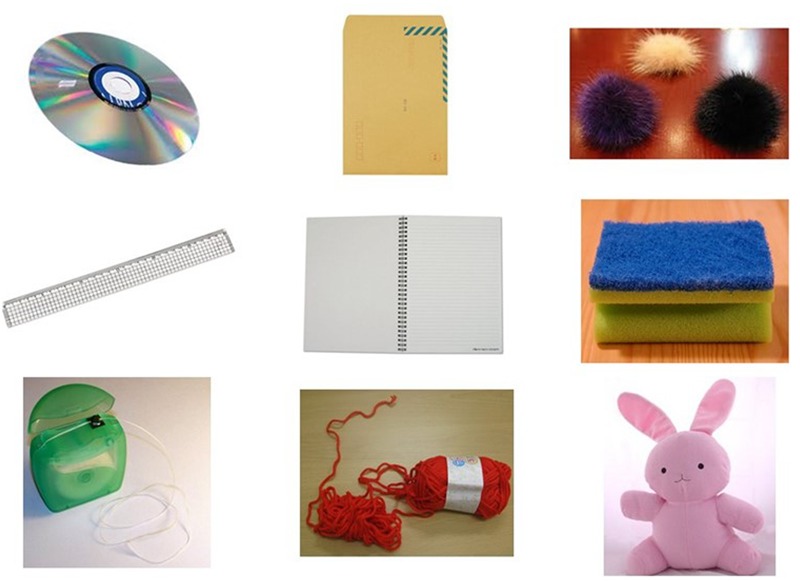
Images of objects used in the cut-able-with/mop-up-with affordance pair block of Experiment 1. Three objects (CD, plastic rule, and dental floss) with cut-able-with affordance only **(Left)**; three objects (envelope, notebook, and knitting ball) with both cut-able-with and mop-up-with affordances **(Middle)**; three objects (fur ornament, kitchen sponge, and bunny doll) with mop-up-with affordance only **(Right)**.

Color images of 27 household items served as the stimuli for the present experiment and are listed in **Table 1**. All images were sized to 600 × 800 pixels and presented on a 15-inch laptop with a pixel resolution of 1024 H × 760 V. Examples are shown in **Figure 1**. The presentation of stimuli was controlled by DirectRT (Jarvis, 2012), which also recorded responses and measured accuracy and reaction times of the responses. Participants viewed the display binocularly at a distance of approximately 50 cm.

**Table 1:** Stimuli used in Experiment 1.

O_aff1_	O_aff1,2_	O_aff2_
**Scoop-with**		**Pierce-with**
Shoes	Kettle	Pencil
Jam jar	Tea scoop	Screwdriver
Bowl	Spoon	Chopsticks
**Pour-in-able**		**Stretchable**
Wooden plate	Rubber glove	Ankle protector
Plastic gum container	Rubber balloon	Stockings
Bottle cap	Swimming goggles	Cloth headband
**Cut-able-with**		**Mop-up-with**
Compact disk (CD)	Envelope	Fur ornament
Plastic ruler	Notebook	Kitchen sponge
Dental floss	Knitting ball	Cotton (bunny) doll

*O_*aff 1*_, O_*aff 2*_ = objects with only one affordance; O_*aff 1, 2*_ = objects with both affordances.*

Each artifact exhibited its designed affordance clearly. Three pairs of affordances were used: (a) scoop-with/pierce-with; (b) pour-in-able/stretchable; (c) cut-able-with/mop-up-with.^5^ As shown in **Table 1**, the nine objects selected to evaluate each affordance pair were divided into three different classes (cf. Ye et al., 2009): O_aff1_ had only the first affordance (e.g., scoop-with) but not the second affordance (e.g., pierce-with); O_aff2_ had only the second affordance but not the first; O_aff1,2_ had both affordances (e.g., scoop-with and pierce with).

### Design

The experiment consisted of three randomized blocks of 108 trials with each block divided by paired affordances (see **Table 1**). Each block was further divided into two half-blocks with each half-block consisting of nine images of objects having either one or both of the paired affordances, that is, three images of objects with the first affordance (O_aff1_), three with the second affordance (O_aff2_), and three having both affordances (O_aff1,2_) (see **Figure 1** for the images of objects that made up the cut-able-with/mop-up-with affordance pair block). Each object appeared twice in each half-block for a total of 18 trials.

Trials were initiated when the participant pressed the space bar, triggering the appearance of a fixation point in the center of the display for 1000 ms, followed by the image of an object presented on a white background. Upon appearance of the object, participants were asked to press as fast and accurately as possible one of the two response keys (left/right shift keys) corresponding to the object’s affordance.

The two response keys were counter-balanced across participants. Thus, for each pair of affordances, an affordance was assigned to one response key, whereas the other affordance was assigned to the other response key, which was repeated once for a total of 18 trials. Then, the same 18 trials were repeated once again, this time by reversing the response keys.^6^ Thus, the 36 randomized trials comprising each block were produced by a 3 (Object Class: O_aff1_, O_aff2_, O_aff1,2_) × 3 (Object) × 2 (Order of Presentation) × 2 (Repetition) scheme.

### Procedure

Participants were tested individually. They were told that household items can be used to perform various functions other than their typical (designed) function. As an example, the experimenter demonstrated that a paper cup designed to contain liquid could be also serve as a candle holder. Using this example, participants were told that their task was to identify one of two actions that could be performed using the displayed object by pressing the corresponding response key.

After 18 trials, participants repeated the same procedure with the response keys reversed for the paired affordances. Prior to the initiation of each half-block, instructions were displayed in text on the computer screen informing participants about the target functions of the displayed objects and the matching response keys.

The pair of affordances examined in each block was explained verbally using the basic descriptions of Ye et al. (2009), but with a slight modification to fit to the Korean culture (see their Appendix A, pp. 213–214).^7^

A 24-trial stimulus–response compatibility task preceded the experiment to allow participants to become familiar with the experimental setup. Displays were made of a red circle (8 cm in diameter) that appeared either to the right or left from the center. For the first 12 trials participants were told to press the spatially congruent response keys (i.e., the right shift key to the right dot and the left shift key to the left dot) as fast and accurately as possible, whereas for the last 12 trials they were told to press the spatially incongruent response keys to the red dot.

A practice session was built using representative objects for each affordance pair block: a gourd dipper for scoop-with-able vs. a nail for pierce-with-able; a mug for pour-in-able vs. rubber bands for stretchable; and a plastic knife for cut-with-able vs. a towel for mop-with-able. The two objects of each affordance pair appeared twice in a practice set. Each practice set was repeated until the participant demonstrated that she fully understood the procedure. A 4-trial practice session preceded each block of the experiment and was repeated in the middle of the block after reversing the response keys. The arrangement of the response keys for each practice set matched that for the 18 trials administered in the ensuing half-block experiment.

Feedback was not provided during the experiment. After two 18-trial sets with a pair of affordances were completed, a short break was given before proceeding to the next block.

### Data Analysis

Performance of the two groups was compared in terms of accuracy and reaction time. Note that the 36 trials comprising each block were produced by a 3 (Object Class: O_aff1_, O_aff2_, O_aff1,2_) × 3 (Object) × 2 (Order of Presentation) × 2 (Repetition) scheme. For each object class, there were three instances of objects. However, there were no systematic relationships among the corresponding objects across the three object classes. The object classes simply were composed of representative objects whose properties were classified as a certain affordance type. In addition, for the class of objects with both affordances (i.e., O_aff1,2_), whichever way they were identified, they were identified accurately. This object class was excluded from the analyses. Thus, for both accuracy and reaction time analyses, responses were collapsed across object and results entered into a 2 (group) × 2 (Object Class: O_aff1_, O_aff2_) × 2 (Order of Presentation) mixed design analysis of variance (ANOVA) with Group as between-subjects factor, and Object Class and Order of Presentation as within-subjects factors.^8^

### Results and Discussion

**FIGURE 2:**
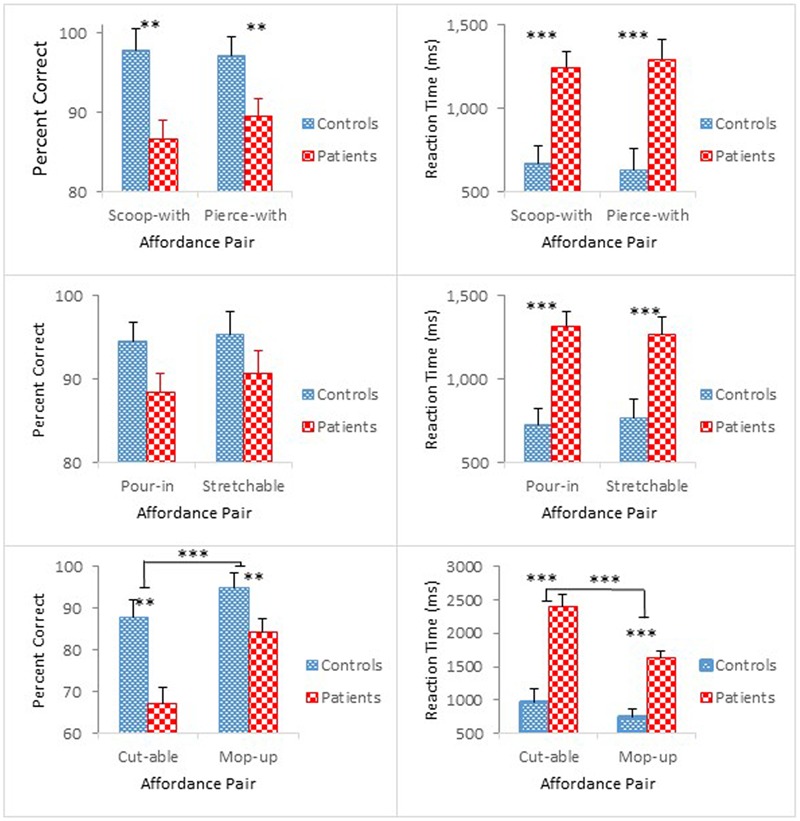
Mean percent correct **(Left)** and mean reaction time **(Right)** (with standard error bars) for healthy controls and schizophrenia participants as a function of paired affordances in Experiment 1. ^∗∗^*p* < 0.01, ^∗∗∗^*p* < 0.001.

**Figure 2** compares group performance in terms of mean percent correct (left panel) and mean reaction time (right panel) as a function of the paired affordances [scoop-with/pierce-with affordance pair (top); pour-in-able/stretchable affordance pair (middle); cut-able-with/mop-up-with affordance pair (bottom)]. Participants with schizophrenia made more errors and took longer to respond than controls. The results of the accuracy analysis for each of the paired affordances are presented first, followed by the results of the reaction time analysis.

#### Accuracy

Overall, schizophrenia participants were less accurate than controls. The ANOVA revealed a main effect of Group for scoop-with/pierce-with affordance pair, *F*(1,47) = 9.00, *p* < 0.01, $\eta_{p}^{2}$ = 0.16. For the pour-in-able/stretchable affordance pair, the effect of Group was marginally significant, *F*(1,47) = 3.80, *p* = 0.057, $\eta_{p}^{2}$ = 0.08. For the cut-able-with/mop-up-with affordance pair, however, the ANOVA, not only revealed a main effect of Group, *F*(1,47) = 18.69, *p* < 0.01, $\eta_{p}^{2}$ = 0.20, but also confirmed a main effect of Object Class, *F*(1,47) = 18.28, *p* < 0.001, $\eta_{p}^{2}$ = 0.28. Apparently, identifying cut-able-with objects was more difficult than identifying mop-up-with objects, both for schizophrenia participants (67% vs. 84%), and for controls (88% vs. 95%).

In addition, the ANOVAs confirmed main effects of Presentation Order for the scoop-with/pierce-with affordance pair, *F*(1,47) = 8.27, *p* < 0.01, $\eta_{p}^{2}$ = 0.15, and for the pour-in-able/stretchable affordance pair, *F*(1,47) = 7.04, *p* < 0.01, $\eta_{p}^{2}$ = 0.13, but a significant interaction between Order and Object Class for the cut-able-with/mop-up-with affordance pair, *F*(1,47) = 6.07, *p* < 0.05, $\eta_{p}^{2}$ = 0.11. Whereas identifying the cut-able-with objects remained difficult throughout the two half-blocks (76% vs. 78%), identifying the mop-up-with objects showed a response pattern similar to those observed in the other affordance pair blocks with performance degradation in the second half-block (94% vs. 85%). Overall, accuracy was worse in the second half-block for both schizophrenia participants and controls.

#### Reaction Time

Schizophrenia participants exhibited longer reaction times than controls. The ANOVA for each affordance pair further confirmed these group differences [*F*(1,47) = 17.65, *p* < 0.001, $\eta_{p}^{2}$ = 0.27 for scoop-with/pierce-with affordance pair; *F*(1,47) = 18.30, *p* < 0.001, $\eta_{p}^{2}$ = 0.28 for pour-in-able/stretchable affordance pair; and *F*(1,47) = 34.17, *p* < 0.001, $\eta_{p}^{2}$ = 0.42 for cut-able-with/mop-up-with affordance pair].

The ANOVA for pour-in-able/stretchable affordance pair also revealed a significant main effect of Presentation Order, *F*(1,47) = 16.03, *p* < 0.001, $\eta_{p}^{2}$ = 0.25, and a Presentation Order by Group interaction, *F*(1,47) = 4.17, *p* < 0.05, $\eta_{p}^{2}$ = 0.08. Both groups responded faster during the second half-block [0.82 s vs. 0.68 s for controls; 1.51 s vs. 1.07 s for schizophrenia participants]. However, the faster reaction time was statistically significant only for schizophrenia participants, *F*(1,47) = 19.47, *p* < 0.001.

For the cut-able-with/mop-up-with affordance pair, the ANOVA further revealed main effects of Order, *F*(1,47) = 15.77, *p* < 0.001, $\eta_{p}^{2}$ = 0.25, and Object Class, *F*(1,47) = 20.80, *p* < 0.001, $\eta_{p}^{2}$ = 0.31, which also interacted with each other, *F*(1,47) = 18.51, *p* < 0.001, $\eta_{p}^{2}$ = 0.28. Moreover, Order and Object Class both interacted with Group [*F*(1,47) = 4.32, *p* < 0.05, $\eta_{p}^{2}$ = 0.08; *F*(1,47) = 6.34, *p* < 0.05, $\eta_{p}^{2}$ = 0.12, respectively], and their 3-way interaction also reached statistical significance, *F*(1,47) = 7.53, *p* < 0.01, $\eta_{p}^{2}$ = 0.14. With respect to the Group and Object Class interaction (bottom right panel of **Figure 2**), although both groups took longer to identify the cut-able-with affordance than the mop-up-with affordance [0.97 s vs. 0.75 s for controls; 2.39 s vs. 1.63 s for schizophrenia participants], the difference in reaction time between these two classes of objects reached statistical significance only for schizophrenia participants, *F*(1,47) = 26.68, *p* < 0.001. Results of the accuracy analysis showed that both controls and schizophrenia participants performed poorly with cut-able-with. This condition was particularly problematic for schizophrenia participants, inducing even longer reaction times. However, the reaction time in this condition was shortened in the second half-block (3.14 s vs. 1.64 s) to a level comparable to the other object class, i.e., mop-up-with (1.64 s vs. 1.52 s), and is the source of the three-way interaction among these variables.

It is of interest to note that accuracy degraded in the second half-block, whereas reaction time decreased in the second half-block for both controls and schizophrenia participants, especially for the pour-in-able/stretchable and cut-able-with/mop-up-with affordance pairs. These two patterns of results are rather contradictory with degraded accuracy suggesting a fatigue effect whereas decreased reaction time suggesting a practice effect. Further comments on these contradictory effects are reserved until more data become available in Experiment 2. Taken together, the results of this experiment suggest that perceptual capacity to identify affordances is likely compromised in patients with schizophrenia.

## Experiment 2: Single Affordance Detection

Experiment 1 employed a two-choice reaction time task in which an object having one of two different affordances appeared on the screen and participants were asked to identify its affordance by pressing one of the two response keys. However, the objects constituting the stimulus set bore no relationship to the response keys constituting the response set, that is, the stimulus and response were incompatible. These arbitrarily arranged stimulus and response sets may have increased the complexity of the task. Impaired cognition is known to be a core feature of schizophrenia (Fioravanti et al., 2005, 2012; Goff et al., 2011). Therefore, it is reasonable to suspect that task complexity, in conjunction with the cognitive deficits prevalent in schizophrenia patients, may have aggravated schizophrenia participants’ performance, resulting in more errors. Moreover, longer reaction times in schizophrenia patients have been well documented, a phenomenon even recognized by Kraepelin (Gale and Holzman, 2000).

Although the two groups were matched in terms of MMSE scores, it was important to rule out, or at least minimize, the possible effect of task complexity on the degraded performance of schizophrenia patients in Experiment 1. To that end, Experiment 2 employed a simple yes/no task. Rather than requiring the participant to identify the secondary affordances of both objects in each set, i.e., affordance 1 (O_aff__1_), or 2 (O_aff2_), in which each set had a different affordance, participants in Experiment 2 identified only one affordance (i.e., affordance 1, O_aff__1_) and rejected objects with any other affordances (i.e., not affordance 1).

### Participants

Twenty-six community-dwelling schizophrenia outpatients (16 males and 10 females) and 23 healthy control participants (14 males and 9 females) participated in the study. None had participated in Experiment 1. Schizophrenia participants had been diagnosed in accordance with the DSM-IV diagnostic criteria for schizophrenia (American Psychiatric Association, 1994) and were following individual medication regimes. Schizophrenia participants were recruited from local (vocational) rehabilitation centers and mental health clinics. The control participants, who had no known family history of mental health disorder, were recruited from the university community.

For the schizophrenia group, the mean age was 39.1 ± 7.32 years, the mean years of education were 13.5 ± 1.94 years, the mean duration of schizophrenia was 15.8 ± 6.1 years, the mean age of onset was 23.5 ± 3.8 years, and the mean MMSE score was 28.3 ± 1.09 (with 8 minimum scores of 27). For the control group, the mean age was 38.6 ± 6.95 years, the mean years of education were 13.6 ± 1.88 years, and the mean MMSE score was 28.5 ± 1.09. The two groups matched for age, *t*(47) = -0.23, *p* > 0.05, education, *t*(47) = 1.92, *p* > 0.05, and MMSE, *t*(47) = 0.55, *p* > 0.05.

All participants had normal or corrected-to-normal vision. Participants received a nominal ($5) fee for their participation in the experiment.

### Apparatus and Materials

The same apparatus and the same color images of 27 household items used in Experiment 1 were adopted.

### Design

The same three affordance pairs from Experiment 1 were used again. This time, each affordance pair block was repeated once for each separate affordance for a total of six randomized blocks of 36 trials each for O_aff__1_ and O_aff2_. Under this arrangement, the target affordance was the signal, and the non-target affordance was the distractor. As in Experiment 1, each block was further divided into two half-blocks by reversing the response keys after 18 trials.

### Procedure

The procedure used in Experiment 1 was replicated with one exception. This time, participants were asked to press one response key if the displayed object had a specific affordance but the other response key if it did not, doing so as quickly and accurately as possible.

The same 24-trial stimulus–response compatibility task used in Experiment 1 preceded the experiment to familiarize participants with the experimental setup. After 18 trials, the same procedure was repeated with the response keys reversed. Prior to the initiation of each half-block, instructions were displayed in text on the computer screen informing of the target function of the displayed objects and the response keys matching the signal and the distractor, respectively. The same 4-trial practice session used in Experiment 1 preceded each half-block of the experiment.

Feedback was not provided during the experiment. After each block, a short break was given before proceeding to the next block.

### Data Analysis

The 36 trials comprising each block were produced by a 3 (Object Class: O_aff1_, O_aff2_, O_aff1,2_) × 3 (Object) × 2 (Order of Presentation) × 2 (Repetition) scheme. Although there were three classes of objects used in each block, one (i.e., O_aff1_) was the target whereas the other (O_aff2_) served as a distractor. Thus, a hit or a correct rejection was coded as a correct response and a miss or a false alarm was coded as an incorrect response. As in Experiment 1 and for a later comparison with the results of Experiments 1 and 3, responses for the class of objects with both affordances (i.e., O_aff1,2_) were deleted from the analyses. Finally, because participants identified objects with a different affordance in each block, it is possible under the present design to assess performance across different affordances directly. Thus, for both accuracy and reaction time analyses, responses were pulled together from all six blocks after collapsing across object and object class and results entered into a 2 (Group) × 2 (Order of Presentation) × 6 (Affordance) mixed design ANOVA with Group as between-subjects factor, and Order of Presentation and Affordance as within-subjects factors.

### Results and Discussion

**FIGURE 3:**
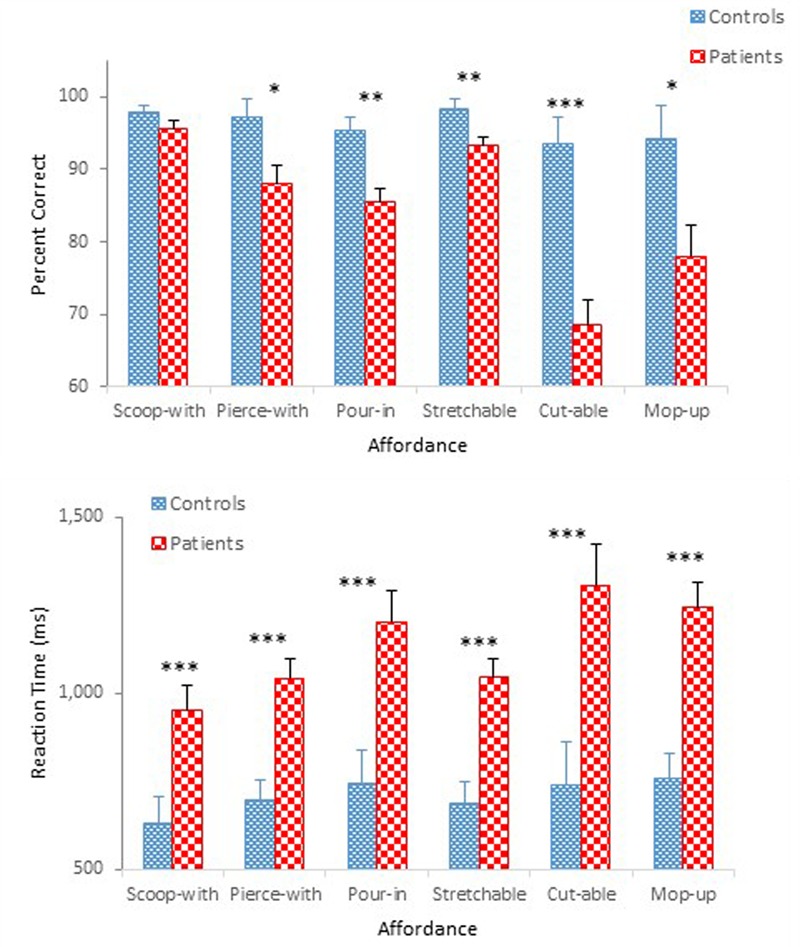
Mean percent correct **(Top)** and mean reaction time **(Bottom)** (with standard error bars) for healthy controls and schizophrenia participants as a function of affordance in Experiment 2. ^∗^*p* < 0.05, ^∗∗^*p* < 0.01, ^∗∗∗^*p* < 0.001.

**FIGURE 4:**
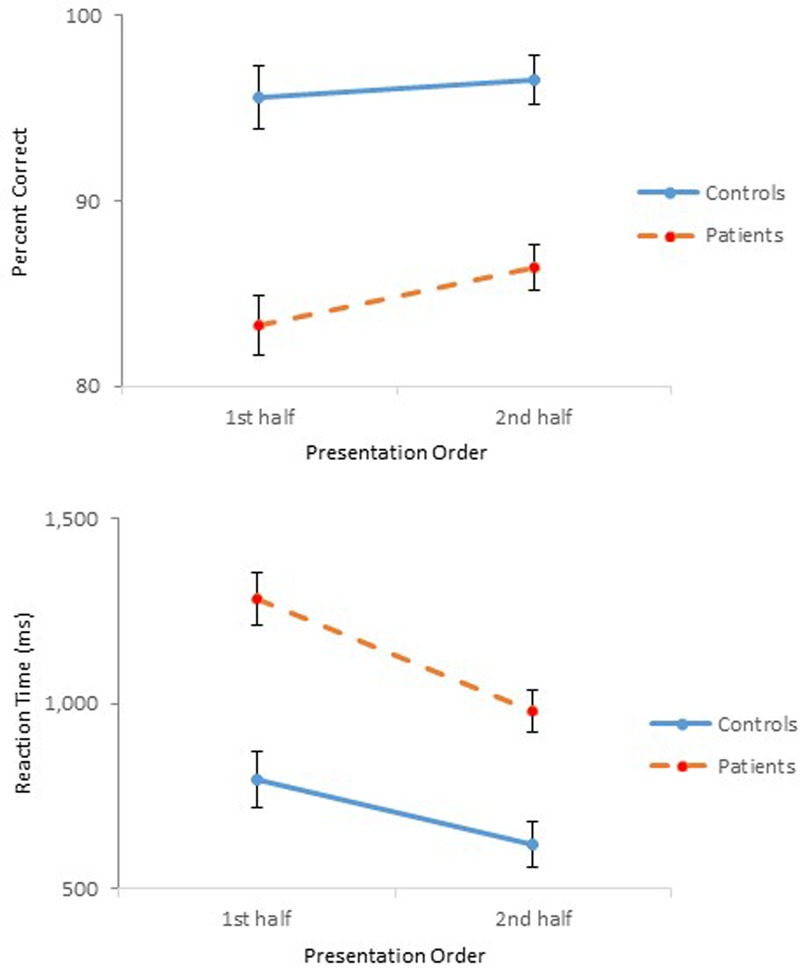
Mean percent correct **(Top)** and mean reaction time **(Bottom)** (with standard error bars) for controls and schizophrenia participants as a function of presentation order in Experiment 2.

**Figure 3** compares performance of the two groups in terms of mean percent correct (top panel) and mean reaction time (bottom panel) as a function of affordance. A mixed design ANOVA on percent correct revealed main effects of Group, *F*(1,47) = 32.74, *p* < 0.001, $\eta_{p}^{2}$ = 0.41, and Affordance, *F*(5,235) = 10.99, *p* < 0.001, $\eta_{p}^{2}$ = 0.19, which was qualified by a significant Group × Affordance interaction, *F*(5,235) = 5.17, *p* < 0.001, $\eta_{p}^{2}$ = 0.10. A simple effects analysis demonstrated a group difference for the pierce-with affordance, *F*(1,47) = 6.34, *p* < 0.05; the pour-in-able affordance, *F*(1,47) = 13.61, *p* < 0.01; the stretchable affordance, *F*(1,47) = 7.43, *p* < 0.01; the cut-able-with affordance, *F*(1,47) = 24.80, *p* < 0.001; and the mop-up-with affordance, *F*(1,47) = 6.81, *p* < 0.05, respectively. However, the effect of Affordance was statistically significant only for schizophrenia participants, *F*(5,43) = 14.48, *p* < 0.001. Schizophrenia participants misidentified the objects with the cut-able-with affordance with particularly high frequency (68%), replicating a pattern observed in Experiment 1. The ANOVA also confirmed a main effect of Presentation Order, *F*(1,47) = 7.87, *p* < 0.01, $\eta_{p}^{2}$ = 0.14 (**Figure 4**). Unlike Experiment 1 in which both groups’ accuracy decreased slightly in the second half-block, the two groups of the present experiment performed slight better in the second half block.

The ANOVA on reaction time demonstrated main effects of Group, *F*(1,47) = 20.91, *p* < 0.001, $\eta_{p}^{2}$ = 0.31, Affordance, *F*(5,235) = 5.64, *p* < 0.01, $\eta_{p}^{2}$ = 0.11, and Presentation Order, *F*(1,47) = 77.16, *p* < 0.001, $\eta_{p}^{2}$ = 0.62. The ANOVA also confirmed significant interactions between Group and Order, *F*(1,47) = 5.79, *p* < 0.05, $\eta_{p}^{2}$ = 0.11 (**Figure 4**), and between Affordance and Order, *F*(5,235) = 3.54, *p* < 0.01, $\eta_{p}^{2}$ = 0.07. With respect to the Group × Order interaction, reaction times were reduced in the second half-block for both controls (0.80 s vs. 0.62 s) and participants with schizophrenia (1.28 s vs. 0.98 s). However, the reduction for participants with schizophrenia appears even steeper. With respect to the Order × Affordance interaction, difference in reaction times across the six affordance objects in the first half-block for both controls and schizophrenia participants, *F*(5,43) = 12.28, *p* < 0.001, became insignificant in the second half-block.

With overall mean accuracy of 84% in Experiment 1 and 85% in Experiment 2, respectively, the effect of task on the performance of the two schizophrenia groups appears negligible. The two control groups also performed similarly with mean accuracy of 95% in Experiment 1 and 96% in Experiment 2, respectively. However, schizophrenia participants in Experiment 2 were more accurate than those in Experiment 1 in identifying the scoop-with affordance (87% in Experiment 1 vs. 96% in Experiment 2), reaching a level comparable to that of controls (98%).

By contrast, the impact of task on reaction time was quite noticeable, particularly for schizophrenia participants. Although the yes/no task shortened reaction time by 0.09 s (0.75 s in Experiment 1 vs. 0.71 s in Experiment 2) for controls, this difference did not reach statistical significance. For the schizophrenia group, by contrast, the yes/no task shortened reaction time by 0.39 s [1.52 s in Experiment 1 vs. 1.13 s in Experiment 2], and the difference was statistically significant at the 0.001 level according to a Tukey *post hoc* test. A one-way ANOVA comparing the four groups’ reaction time data [two control groups of Experiments 1 and 2 and two schizophrenia groups of Experiments 1 and 2] confirmed a main effect of Group, *F*(3,94) = 21.43, *p* < 0.001, $\eta_{p}^{2}$ = 0.41.

The results of Experiment 1 demonstrated the effect of Presentation Order through degraded accuracy (possibly a fatigue effect), but faster reaction time (possibly a practice effect) in the second-half block, not only for schizophrenia participants but also for controls. The same Order effect was observed in the present experiment for both controls and schizophrenia participants. This time, however, both groups performed more accurately and faster in the second half-block (**Figure 4**), although the improved performance of schizophrenia participants did not achieve that of controls. When performance of the two groups during the second-half block was compared using a 2 (Group) × 6 (Affordance) mixed design ANOVAs, the ANOVA on percent correct confirmed main effects of Group, *F*(1,47) = 31.21, *p* < 0.0001, $\eta_{p}^{2}$ = 0.40, and Affordance, *F*(5,235) = 10.46, *p* < 0.0001, $\eta_{p}^{2}$ = 0.18, and a significant Group × Affordance interaction, *F*(5,235) = 4.08, *p* < 0.01, $\eta_{p}^{2}$ = 0.08, the same effects observed when the data from both blocks were analyzed. Interestingly, however, in the combined analysis, group difference disappeared only for the scoop-with affordance. This time, however, performance difference of the two groups for the stretchable affordance also became indistinguishable, *F*(1,47) = 3.67, *p* = 0.06. The ANOVA on reaction time, however, confirmed only a main effect of Group, *F*(1,47) = 17.84, *p* < 0.0001, $\eta_{p}^{2}$ = 0.28. Schizophrenia participants took longer to respond (0.98 s) than controls (0.62 s).

Overall, the impact of the simplified yes/no task on accuracy was limited, although it facilitated the identification of one particular affordance, i.e., the scoop-with affordance. The impact on reaction time became more apparent. Still schizophrenia participants took longer to respond than controls, even considering a likely practice effect that further decreased reaction time in the second-half block. It appears, therefore, that a yes/no task facilitated schizophrenia participants’ performance to some degree, but not enough to undermine our contention that perceptual capacity to identify affordances is likely impaired in patients with schizophrenia.

With respect to the main effect of Affordance in which certain affordances, e.g., the scoop-with affordance, were better identified than other affordances, it is possible that the degree to which the three objects chosen for each affordance represent the target affordance in the present study may have been different. That is, some objects may have been better exemplars of a particular affordance than others (see Ye et al., 2009, for a discussion on this issue). Of interest is the fact that the cut-able-with affordance, which caused more errors than other affordances for both controls and participants with schizophrenia in Experiment 1, became non-distinct for controls but remained problematic for participants with schizophrenia in Experiment 2. Another possibility is that the degree to which human observers are attuned to each affordance may not be the same. Thus, certain affordances are more salient than others. More research is needed to corroborate these possibilities.

## Experiment 3: Physical Property Detection

To date, research on cognitive impairment in schizophrenia has focused primarily on attention, memory, and executive functioning (Fioravanti et al., 2005, 2012; Goff et al., 2011). Although less well known, evidence is mounting demonstrating that schizophrenia also disrupts visual processing, causing deficits in a variety of visual functions (see Butler et al., 2008; Silverstein et al., 2015, for reviews). Thus, another possibility still exists implicating visual processing deficits in schizophrenia as the cause of poor performance observed in participants with schizophrenia. Experiment 3 was conducted to rule out this possibility.

Using the yes/no task employed in Experiment 2 and the same images of objects as in the previous experiments, participants were asked to identify objects’ physical properties (e.g., shape, color, or material composition) instead of their functions. Specifically, participants were asked to judge whether the displayed image of the object(s) contained a certain color (e.g., pink), a certain shape feature (e.g., a right angle), or a certain material (e.g., fabric). Six physical properties—two colors (pink and green), two shapes (right angle and circle), and two types of material (fabric and wood)—were chosen for this purpose. As in Experiment 2, the experiment was composed of six randomized blocks with each block assessing each single physical property separately.

### Participants

Experiment 1’s participants also participated in Experiment 3.

### Apparatus and Stimuli

The same apparatus and stimuli used in the previous experiments were used.

### Design

The experiment was conducted in six randomized blocks with each block comprising a pair of physical properties. The paired properties were: (a) pink/right angle, (b) fabric/circular, (c) green/wooden-material. As in Experiment 2, each of these three blocks was repeated once for a separate physical property with the objects from the target property as signals, and the objects from the non-target property as distractors. Each property was represented by three objects for a total of six objects comprising each block. The objects chosen for each physical property are listed in **Table 2**. The six objects were presented twice producing a total of 12 trials, which were repeated after reversing the response keys. Thus, the 24 randomized trials constituting each block were produced by a 2 (Object Class: O_phys1_, O_phys2_) × 3 (Object) × 2 (Order of Presentation) × 2 (Repetition) scheme.

**Table 2:** Stimuli used in Experiment 3.

Physical properties	Objects		
Pink	Cotton (bunny) doll	Cloth headband	Rubber glove
Right angle	Plastic ruler	Notebook	Envelope
Fabric	Stocking	Kitchen sponge	Ankle protector
Circle	Plastic gum container	Compact disk (CD)	Jam jar
Green	Bottle cap	Rubber balloon	Dental floss
Wood	Wooden plate	Tea scoop	Pencil

### Procedure

The procedure used in Experiment 2 was replicated with one exception. Participants were asked to press one response key if the displayed object had a specific physical property and the other response key if it did not as fast and accurately as possible.

As in the previous experiments, a 2-trial practice session preceded each block of the experiment and occurred in the middle of the block after reversing the response keys. The arrangement of the response keys of each practice set matched that of the 12 trials administered in the ensuing half-block experiment. The representative images that were used to produce practice trials in each block were pink roses (pink), a window (right angle), cloth (fabric), a coin (circle), a green bell pepper (green), and a wooden toy dog (wood).

After 12 trials, participants repeated the same procedure with the response keys reversed. Prior to the initiation of each half-block, instructions were displayed in text on the computer screen informing participants about the target property in the displayed objects and the response keys matching the signal and the distractor, respectively.

Feedback was not provided during the experiment. After two 12-trial sets with a physical property were completed, a short break was given before proceeding to the next block.

### Data Analysis

The 24 trials comprising each block were produced by a 2 (Object Class: O_phys1_, O_phys2_) × 3 (Object) × 2 (Order of Presentation) × 2 (Repetition) scheme. As in Experiment 2, of the two classes of objects constituting each block, one was the target whereas the other served as a distractor. Thus, a hit or a correct rejection was coded as a correct response and a miss or a false alarm was coded as an incorrect response. Again as in Experiment 2, responses were combined from all six blocks after collapsing across object and object class and results entered into a 2 (Group) × 2 (Order of Presentation) × 6 (Physical Property) mixed design ANOVA for accuracy and reaction time analyses, separately.

### Results and Discussion

**FIGURE 5:**
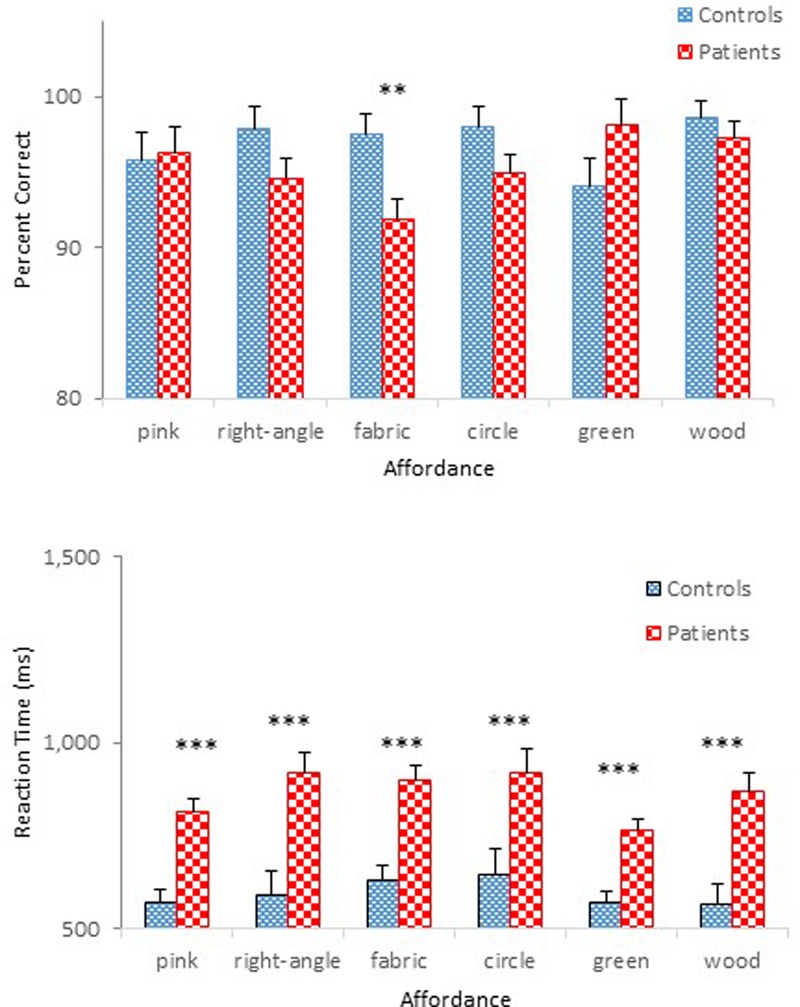
Mean percent correct **(Top)** and mean reaction time **(Bottom)** (with standard error bars) for controls and schizophrenia participants as a function of physical property in Experiment 3. ^∗∗^*p* < 0.01, ^∗∗∗^*p* < 0.001.

Mean percent correct (top panel) and mean reaction time (bottom panel) of two groups are presented as a function of physical property in **Figure 5**. Unlike the previous experiments, schizophrenia participants were extremely accurate in identifying objects’ physical properties with an overall mean of 95 %, a level comparable to that of controls (97%). Indeed, the ANOVA confirmed that this difference was not significant, *F*(1,47) = 1.69, *p* = 0.20. The Group × Physical Property interaction, however, reached significance, *F*(5,235) = 3.09, *p* < 0.05, $\eta_{p}^{2}$ = 0.06. A simple effects analysis revealed that schizophrenia participants made more errors in identifying the fabric objects (92%), causing this interaction, *F*(1,47) = 8.30, *p* < 0.01. Further inspection of data revealed that it was the kitchen sponge that caused most confusion for schizophrenia participants. The mean percent correct of the two groups for each object used in the fabric objects condition is shown in **Table 3**. The kitchen sponge image used in the experiment showed two layers, with one that was made of a synthetic fabric-like material (see the middle right panel in **Figure 1**). Note that today most kitchen sponges, including the image that was used in the present experiments, are made from foamed plastic polymers. In hindsight, we should have used a more intuitive fabric object. Interestingly, the control group identified the kitchen sponge as a fabric object as accurately (96%) as the other two fabric objects. It is not clear, therefore, why the kitchen sponge was particularly problematic for schizophrenia participants.

**Table 3:** Mean percent correct (with standard deviation) for controls and schizophrenia participants in the fabric objects condition of Experiment 3.

Object	Controls	Patients
Stocking	98 (6)	97 (6)
Kitchen sponge	96 (6)	86 (6)
Ankle protector	98 (9)	93 (9)

By contrast, schizophrenia participants took longer to respond (0.86 s) than controls (0.60 s), a pattern replicating that observed in the previous experiments. An ANOVA on reaction time confirmed main effects of Group, *F*(1,47) = 23.63, *p* < 0.001, $\eta_{p}^{2}$ = 0.34, Physical Property, *F*(5,235) = 3.39, *p* < 0.01, $\eta_{p}^{2}$ = 0.07, and Presentation Order, *F*(1,47) = 18.78, *p* < 0.001, $\eta_{p}^{2}$ = 0.29. The interaction between Physical Property and Order also reached statistical significance, *F*(5,235) = 3.32, *p* < 0.01, $\eta_{p}^{2}$ = 0.07. The effect of Order was significant for the right angled objects, *F*(1,47) = 7.64, *p* < 0.01; the fabric objects, *F*(1,47) = 7.69, *p* < 0.01; and the circular objects, *F*(1,47) = 10.03, *p* < 0.01. However, the difference in reaction times across the six physical propertied objects in the first half-block for both controls and schizophrenia participants, *F*(5,43) = 3.88, *p* < 0.01, disappeared in the second half-block, *F* < 1, ns.

**FIGURE 6:**
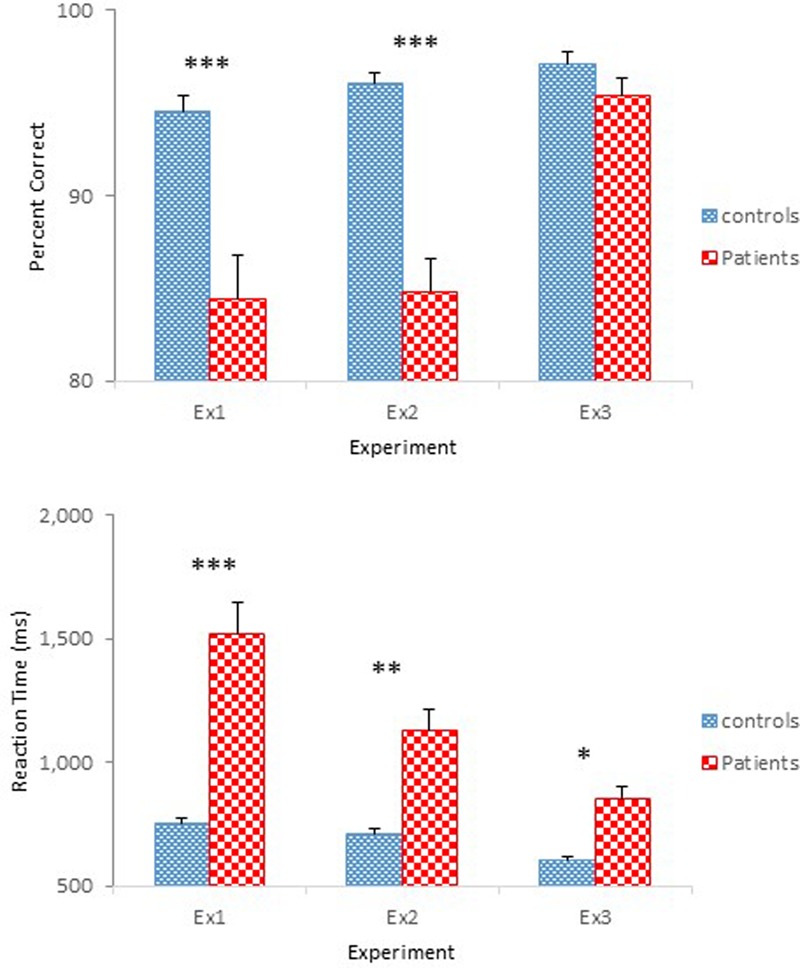
Mean percent correct **(Top)** and mean reaction time **(Bottom)** (with standard error bars) for controls and schizophrenia participants in Experiments 1 through 3. ^∗^*p* < 0.05, ^∗∗^*p* < 0.01, ^∗∗∗^*p* < 0.001.

The results of Experiment 3 were straightforward. Schizophrenia participants performed as accurately as controls in identifying physical properties contained in the objects, but showed slower reaction times. However, schizophrenia participants responded more quickly than in the previous experiments. **Figure 6** shows performance of the two groups across the three experiments. Improved performance by the schizophrenia participants in Experiment 3 is quite evident. Two one-way ANOVAs were conducted to compare the performance of the four groups participated in Experiments 2 and 3, one on percent correct and the other on reaction time. Both ANOVAs confirmed main effects of Group [*F*(3,94) = 26.60, *p* < 0.001, $\eta_{p}^{2}$ = 0.46, for accuracy; *F*(3,94) = 17.97, *p* < 0.001, $\eta_{p}^{2}$ = 0.36, for reaction time]. A Tukey *post hoc* test on percent correct revealed that performance of the Experiment 2 schizophrenia group differed significantly from that of the other three groups, all at the.001 level. Importantly, the Experiment 3 schizophrenia group did not differ from the two control groups of Experiments 2 and 3. The same test on reaction time failed to find a significant difference between the two control groups, but found significant differences between the Experiment 2 schizophrenia group and the other three groups, all at the 0.001 level. Importantly, the same test failed to reveal a significant difference between the Experiment 3 schizophrenia group and the Experiment 2 control group.

Still, schizophrenia participants responded slower than controls. However, the level of performance demonstrated by schizophrenia participants (0.86 s) was comparable to that of the schizophrenia patients group (0.84 s) reported in Sevos et al. (2013). Interestingly, their control group performed comparably to that demonstrated by the controls of the present experiment (0.61 s vs. 0.60 s). Also notable in the Sevos et al. (2013) study was equally accurate level of performance by their two groups (91% controls and 90% patients), a similar pattern observed in the present experiment. Thus, the longer reaction times may have been the result of the impaired reaction time mechanism in schizophrenia patients, consistent with existing literature (Gale and Holzman, 2000, for review).

Experiment 3 was conducted to examine whether schizophrenia participants’ performance in the previous experiments was somehow affected by visual processing impairments of schizophrenia reported in the literature (Butler et al., 2008; Silverstein et al., 2015). For this purpose, we used the same color images of the objects used in the previous experiments. Accurate identification of the physical properties of the objects demonstrated by schizophrenia participants in the present experiment is assuring providing a strong basis to rule out possible influence of the reported visual deficits in schizophrenia on the task employed in the present study. Taken together, the degraded performance demonstrated by the schizophrenia participants in the two previous experiments is likely due to an impaired capacity to perceive affordances.

## General Discussion

The present study was inspired by phenomenological psychopathologists who conceptualize schizophrenia as a self-disorder (Parnas, 2000, 2003, 2011, 2012; Stanghellini and Ballerini, 2002; Sass, 2003a,b, 2014; Sass and Parnas, 2003, 2007; Cermolacce et al., 2007; Uhlhaas and Mishara, 2007; Stanghellini, 2009; Parnas and Sass, 2010; Raballo et al., 2011; Nelson et al., 2014). Particularly disturbed is the minimal self, or ipseity, thought to grant a pre-reflective (i.e., non-conceptual) and primitive level of subjective awareness. Engendered by two complementary processes, hyperreflexivity and diminished self-affection, these distortions deprive a schizophrenia person of the first-person perspective of one’s own experience and the sense of immersion in the world (Gallagher, 2000; Sass and Parnas, 2007; Zahavi, 2005; Nelson et al., 2014). Symptoms manifested include “depersonalization, diminishing sense of existing as a bodily subject, distortions of first-person perspective with weakened sense of ‘mineness’ of the field of awareness (thoughts, sensations, etc.), diminished sense of coherence and consistency in fundamental features of self (e.g., sense of anonymity, identity confusion, etc.), and disturbed self-other/self-world boundaries” (Nelson et al., 2014, p. 479). The consequence is that the schizophrenia person becomes disembodied, living as a mere spectator of his own perceptions, actions, and thoughts (Stanghellini, 2009, p. 58).

Despite recent interest in the phenomenological perspective on schizophrenia, behavioral research demonstrating these alterations in the subjective aspects of self-experience in schizophrenia is limited (cf. Sevos et al., 2013). The present study was conducted to fill that void. Three experiments investigated schizophrenia patients’ affordance perception. Affordance is arguably the central concept of perception scientist James J. Gibson’s ecological approach to perception and action. Referring to environmental properties taken with reference to an animal, affordances are intrinsic properties of objects and events by virtue of their makeup with respect to a particular perceiver-actor. As such, affordances provide behavioral opportunities to the animal. Importantly, based on the principle of reciprocity, an affordance binds the reciprocal pairs of animal and environment, perception and action, (subjective) self and (objective) world, into a dynamic whole. Thus, it is affordance that sets into motion the dynamic interplay between reciprocally coupled processes. Following the contention of phenomenological psychopathologists, if selfhood is indeed disturbed in schizophrenia, it is likely that this dynamic interplay would be perturbed and schizophrenia patients’ capacity to perceive affordances severely compromised. The present results suggest that affordance perception may indeed be compromised in schizophrenia patients.

In Experiment 1, employing a two-choice reaction time task, participants judged secondary affordances of household items, that is, the non-designed functions of objects (e.g., whether a pencil, primarily designed as a writing instrument, can be used to tear a plastic bag filled with sand). Schizophrenia participants made more identification errors (84% vs. 95%) and took longer to respond (1.52 s vs 0.75 s) than healthy controls. Considering the cognitive deficits reported in schizophrenia (Fioravanti et al., 2005, 2012; Goff et al., 2011), Experiment 2 attempted to minimize task complexity by employing a yes/no task. The simplified task facilitated the identification of one particular affordance, but had a greater impact on reaction time, particularly for schizophrenia participants inducing, on average, a 0.39 s reduction. Still, schizophrenia participants’ performance fell far below that of controls. To further rule out the possible contribution of the reported visual abnormalities in schizophrenia on the performance observed in the first two experiments, Experiment 3 had participants identify physical properties (e.g., color, shape, material composition) of the objects using the same images of objects used in the previous experiments. Schizophrenia participants were as accurate as controls and responded faster than in the previous experiments. Taken together, the present findings suggest that the capacity to perceive affordances is likely impaired in people with schizophrenia.

To date, ample evidence is available demonstrating human observers’ capacity to perceive a variety of affordances (Fajen et al., 2008, for review). By perceiving affordances, one becomes aware of functionally significant properties of the surrounding environment. Founded on the cyclic processes of perceiving and acting, the awareness arising from perceptual encounters with the world is primitive and pre-reflective and, at the same time, direct and unmediated. Such immediacy renders the animal’s encounters with the environment to be automatic and habitual, and the barrier separating the self and the world to disappear (Heft, 2003). The animal becomes “immersed in situated doing and being” (Heft, 2003, p. 151), that is, it is embodied.

Alternatively, Heft (2003) contends, that one can maintain contact with the world by engaging in what he refers to as the “second-order mode of knowing.” To engage in this mode of knowing, one has to “step outside of the ongoing flow of immediate perception–action awareness by reflecting on the things of the environment; that is, [one has to] shift the necessarily selective character of [one’s] attentional focus from experiencing the immediate flow of events to experiencing the experience and, in doing so, isolate particular portions of immediate experience, holding them in awareness for analysis, categorization, or other second-order or indirect acts of cognition. Accompanying these acts of reflexivity is a comparative heightening of awareness, as entities in experience are momentarily lifted out of the perceptual flow for closer scrutiny” (p. 151). Consequently, one can experience physical objects, but only as neutral things devoid of any psychological values. Not being able to relate to these objects (i.e., not being able to perceive affordances), one is not “drawn toward them or repelled by them for any intrinsic qualities they possess” (Heft, 2003, p. 151). Thus, one becomes detached from the world.

Heft’s (2003) remark was intended to contrast Gibson’s ecological framework with the traditional indirect physicalist framework of cognition. He conjectures mental states that can arise when one engages in the “second-order mode of knowing.” Still, one cannot help but notice the striking resemblance between these mental states and those of schizophrenia patients portrayed by phenomenological psychopathologists. However, it may be premature to speculate how the impaired capacity to perceive affordances of schizophrenia patients may contribute to their being disembodied, the fundamental feature of schizophrenia patients’ experience. Further pursuing this issue is left for future research.

## Conclusion

The present study empirically explored schizophrenia patients’ affordance perception. Affordance, as Gibson (1986/1979) pointed out, is a concept that “cuts across the dichotomy of subjective–objective” (p. 129). If distortions of selfhood lie at the core of schizophrenia as phenomenological psychopathologists contend, the capacity to perceive affordances should be equally disturbed, the question addressed in this study. The present findings appear to corroborate this conjecture.

However, we also acknowledge several limitations of the study. First, heterogeneity in manifested symptoms has been well recognized as the hallmark of schizophrenia (Arango and Carpenter, 2011). Of these symptoms, motor abnormalities (e.g., abnormal involuntary movements, parkinsonism, neurological soft signs, and psychomotor slowing) have been known as one characteristic of this disorder (Walther and Strik, 2012, for review). Recent findings in affordance perception research demonstrate that perceived surface layout changes in accordance with changes in one’s action capabilities (Proffitt, 2006, for review). It is conceivable, then, that schizophrenia participants’ altered motor capacity could have affected their capacity to perceive affordances in the present study. As it stands, this possibility cannot be ruled out in interpreting the present findings, and thus is left for future consideration.

Second, Experiment 3 was conducted to rule out the possible influence of the reported visual anomalies in schizophrenia on the processing of the stimuli used in the present experiments. The comparable accuracy of schizophrenia participants to that of controls provided a strong basis to rule out this possibility. However, a growing number of studies has reported impaired perceptual organization as another symptom manifestation of schizophrenia (Phillips and Silverstein, 2003; Uhlhaas and Silverstein, 2005). Thus, while the accurate performance of schizophrenia patients in Experiment 3 provides evidence for accurate detection of individual features, it leaves open the possibility that the degraded performance of schizophrenia patients in Experiments 1 and 2 may have been due to impaired capacity to group stimulus elements into coherent object representations.

Third, the MMSE we used to screen participants for cognitive impairment does not include cognitive deficits such as attention, working memory, and executive functioning (Fioravanti et al., 2005, 2012; Goff et al., 2011) which may have affected the performance of schizophrenia participants. These issues too will require further research.

Finally, for methodological reasons stated earlier, the present study employed computer-based, choice reaction time tasks with images of the objects as stimuli. The question can be raised, then, whether affordances conveyed by images of objects are the same as those conveyed by real objects. This issue intrigued Gibson (1971, 1986/1979) throughout his career. The conclusion Gibson ultimately reached was that the information contained in an optic array generated by a picture is the same as that generated by a real object^9^, assuring that affordance perception can be studied using images of objects. Further addressing this issue goes beyond the scope of the present study. In the future, however, we intend to replicate the current study using real objects as done by Ye et al. (2009).

To conclude, it appears that the capacity to perceive affordances is likely impaired in people with schizophrenia. More corroborating evidence is needed to confirm the present findings. Nevertheless, an argument can be made, based on the present findings, that affordance, the central concept in Gibson’s ecological approach to perception and action, can shed further insight into the phenomenological core of schizophrenia, alterations of self, and the accompanying disembodiment, another fundamental feature of schizophrenia. Moreover, assessing one’s capacity to perceive affordances is a measure that can be easily administered as shown in this study. Considering the difficulties in devising scientific methods to probe subjective conscious experience empirically, it is hoped that the present study stimulates further empirical research into affordance perception in schizophrenia to gain greater understanding of this complex disorder.

## Author Contributions

Both authors have made substantial, direct and intellectual contribution to the work, and approved it for publication.

## Conflict of Interest Statement

The authors declare that the research was conducted in the absence of any commercial or financial relationships that could be construed as a potential conflict of interest.

## Footnotes

1 It is important to recognize that Gibson was not the first person who considered environmental features in terms of their functional significance or action capabilities. Phenomenological philosophers such as, Heidegger, Von Uexküll, and Merleau-Ponty took a similarly relational perspective that regards world and individual as an integrated complex system, leading them to develop concepts similar to Gibson’s affordance such as Heidegger’s ‘equipment’ and ‘ready-to-hand,’ Von Uexküll’s ‘Umwelt,’ Lewin’s ‘valence,’ and Koffka’s ‘demand character’ (Sanders, 1993; Kadar and Effken, 1994; Heft, 2001; Withagen et al., 2012; de Haan et al., 2013). Gibson himself acknowledged the ‘demand character’ of Lewin and Koffka as the inspiration for his affordance concept.

2 This remark by Gibson has been controversial generating a series of debates among ecological psychologists with some conjecturing affordances as properties of the environment independent of a perceiver (Turvey, 1992; Reed, 1996; Heft, 2001), and others conjecturing affordances as relations between action capabilities of the animal and physical properties of the environment, (Chemero, 2003; Stoffregen, 2003). Further discussion of this issue, however, is beyond the scope of this study. Interested readers are referred to the studies cited here.

3 In conventional cognitive science, cognition is primarily conceived as a brain function completely detached from the body. By contrast, embodied cognition views that the mind is not only connected to the body but, more importantly, is fundamentally shaped by bodily interactions with the world. While several versions of embodied theory of cognition have been advanced (Wilson, 2002), the usage of embodiment and disembodiment in this context is primarily theoretical. However, as used by phenomenological psychopathologists, disembodiment is used quite literally in characterizing the mental states of patients with schizophrenia. As Stanghellini’s (2009) describes: “The disembodiment of the self, of the self-object relation and of interpersonal relationships all lead back to a kind of world in which the schizophrenia person lives and behaves like a soulless body or a disembodied spirit” (p. 59) or “This radical dualism between a *subject* who’s thinking and an *object* that is conceived of in its pure and simple extensive externalness – pure consciousness and pure materialness – is the fundamental phenomenon of schizophrenic anomalies of embodied self-awareness” (p. 58). Lacking such “embodied self-awareness” creates anomalies with how the individual relates to (and assesses meaning to) objects. Specifically, not recognizing his own body as involved in the world, the schizophrenia patients may no longer recognize the utility of objects—or even their practical meanings.

4 Michaels (1988) was the first to suggest using reaction time to investigate affordances, on the premise that “affordances differ in their abilities to attract attention so that the information specifying some affordances would be faster or easier to detect” (p. 232). Since then, quite a few studies have been conducted employing reaction time to investigate affordance perception (e.g., Tucker and Ellis, 1998; Phillips and Ward, 2002; Symes et al., 2007, to name a few).

5 In selecting the objects used in the present study, we followed the procedure used by Ye et al. (2009). Specifically, all the objects were restricted in size to fit on a tray; but, most importantly, their designed (i.e., intended) functions differed from the non-designed affordances (uses) to be identified by participants in the present study. Unlike Ye et al. (2009), who recruited 20 college students to assess the primary use of each object that was adopted in their study, we recruited three graduate students to carry out the selection procedure. For details of the procedure, see Ye et al.’s (2009) Appendix B, pp. 214–215. Cultural differences necessitated replacing a few objects. For example, instead of a turkey baster we used a fur ornament and a cotton (bunny) doll.

6 For the first 18 trials, half of the participants pressed the left shift key if the displayed object was perceived to have the first affordance (O_aff1_) and the right shift key if it was perceived to have the second affordance (O_aff2_). For the next 18 trials, the response keys were reversed such that participants pressed the right shift key if the displayed object was perceived to have the first affordance (O_aff1_) and the left shift key if it was perceived to have the second affordance (O_aff2_). This arrangement was reversed for the other half of the participants.

7 For the affordance pair scoop-with/pierce-with, a (grocery store) plastic bag filled with sand was shown to the participant. Then, the affordance scoop-with was described as “an object that you could use to scoop sand out of this plastic bag if the bag were torn open”; whereas the affordance pierce-with was described as “an object that you could use to poke a hole or tear this plastic bag filled with sand.” For the affordance pair pour-in-able/stretchable, the affordance pour-in-able was described as “a container or enclosed space into which you can pour a small amount of water so that the water would not leak out”; whereas the affordance stretchable was described as “an object that you can stretch by holding it in your hands and stretching it so that it is noticeably different in length without breaking or tearing the material.” For the affordance pair cut-able-with/mop-up-with, the affordance cut-able-with was described as “something that could be used to cut a birthday cake into small strips”; and the affordance mop-up-with was described as “an object that you could use to help mop up a glass of water that spilled on a table.” Note that Ye et al. (2009) used Play-Doh to describe the affordance cut-able-ness. We replaced the Play-Doh with birthday cake due to Play-Doh’s unfamiliarity to middle aged Koreans.

8 Some psychiatrists of schizophrenia participants did not provide the amount of medication prescribed citing doctor–patient confidentiality. This prevented us from factoring in the amount of medication as a covariate in the analyses. For this reason, caution should be exercised not to overgeneralize the present findings.

9 For Gibson (1971), “*A picture is a surface so treated that a delimited optic array to a point of observation is made available that contains the same kind of information that is found in the ambient optic arrays of an ordinary environment*” (p. 31, italics original).
